# Global optimal eBURST analysis of multilocus typing data using a graphic matroid approach

**DOI:** 10.1186/1471-2105-10-152

**Published:** 2009-05-18

**Authors:** Alexandre P Francisco, Miguel Bugalho, Mário Ramirez, João A Carriço

**Affiliations:** 1 Instituto de Engenharia de Sistemas e Computadores – ID em Lisboa, Rua Alves Redol 9, 1000-029 Lisboa, Portugal; 2 Instituto Superior Técnico, Universidade Técnica de Lisboa, Av. Rovisco Pais, 1049-001 Lisboa, Portugal; 3 Instituto de Microbiologia/Instituto de Medicina Molecular, Faculdade de Medicina, Universidade de Lisboa, Av. Prof. Egas Moniz, 1649-028 Lisboa, Portugal

## Abstract

**Background:**

Multilocus Sequence Typing (MLST) is a frequently used typing method for the analysis of the clonal relationships among strains of several clinically relevant microbial species. MLST is based on the sequence of housekeeping genes that result in each strain having a distinct numerical allelic profile, which is abbreviated to a unique identifier: the sequence type (ST). The relatedness between two strains can then be inferred by the differences between allelic profiles. For a more comprehensive analysis of the possible patterns of evolutionary descent, a set of rules were proposed and implemented in the eBURST algorithm. These rules allow the division of a data set into several clusters of related strains, dubbed clonal complexes, by implementing a simple model of clonal expansion and diversification. Within each clonal complex, the rules identify which links between STs correspond to the most probable pattern of descent. However, the eBURST algorithm is not globally optimized, which can result in links, within the clonal complexes, that violate the rules proposed.

**Results:**

Here, we present a globally optimized implementation of the eBURST algorithm – goeBURST. The search for a global optimal solution led to the formalization of the problem as a graphic matroid, for which greedy algorithms that provide an optimal solution exist. Several public data sets of MLST data were tested and differences between the two implementations were found and are discussed for five bacterial species: *Enterococcus faecium*, *Streptococcus pneumoniae*, *Burkholderia pseudomallei*, *Campylobacter jejuni *and *Neisseria spp.*. A novel feature implemented in goeBURST is the representation of the level of tiebreak rule reached before deciding if a link should be drawn, which can used to visually evaluate the reliability of the represented hypothetical pattern of descent.

**Conclusion:**

goeBURST is a globally optimized implementation of the eBURST algorithm, that identifies alternative patterns of descent for several bacterial species. Furthermore, the algorithm can be applied to any multilocus typing data based on the number of differences between numeric profiles. A software implementation is available at .

## Background

Sequence based typing methods are rapidly becoming the gold standard for epidemiological surveillance with the data generated being also used to study microbial population genetics.

Of the several methods developed, Multilocus Sequence Typing (MLST) [1] has been widely applied to clinically relevant bacterial species, and online databases have been implemented [2-5], facilitating the sharing and analysis of MLST data. The method itself is based on the sequencing of specific regions of (typically) seven housekeeping genes of the microorganism of choice. The selection of housekeeping genes stems from the assumption that they are under moderate to strong purifying selection, and the resulting sequence variation is mostly neutral. Therefore, the accumulation of variation by mutation, in the absence of recombination, occurs approximately linearly with time, and the resulting genetic distance tends to be proportional to the time of divergence between alleles. Those sequences are compared to an allele database for each gene, each unique sequence is assigned a numerical identifier and the combination of alleles at each locus creates an allelic profile. Each unique allelic profile is then converted to a Sequence Type (ST) that unambiguously identifies a clone. The main aim of MLST is to provide a portable, accurate, and highly discriminative typing system and this approach has been used successfully with many different bacteria and other microorganisms.

The first approach to the MLST data analysis for the illustration of the relationships between isolates, was the creation of Unweighted Pair Group Method with Arithmetic mean (UPGMA) dendrograms from distance matrices containing the pairwise differences of allelic profiles [6,7]. This type of cluster analysis presents several advantages, such as the ease of interpretation and the creation of an hierarchical grouping of the isolates, that can provide a global overview of the relatedness of the isolates under study and how the defined clusters are connected to each other. However, the topology of such dendrograms frequently does not reflect the patterns of descent [8]. As discussed by Hall and Barlow [9] bifurcating tree-like representations of the relationships between isolates are frequently misinterpreted as depicting the precise evolutionary history of the isolates and are particularly susceptible to the confounding effects of recombination. A bifurcating tree representation, rather than a graph-based one, may more easily lead to false conclusions about the relationships of the isolates, and the latter should be preferred when recombination is high [10].

Other analyses methodologies focus directly on the sequence data instead of on an allelic profile. Although supported by a large body of work [11], these methods face an even greater challenge of disentangling the role of recombination over mutation. Distance metrics based on the comparison of a simple alignment of concatenated sequences can be greatly affected by a single recombination event that results on multiple nucleotide changes, thereby disrupting the phylogenetic signal. In spite of these caveats, Multilocus Sequence Analysis (MLSA) has been successfully used for the phylogenetic analysis of species relationships, partly because the use of multiple loci helps to circumvent this problem [12,13]. Recently, a new Bayesian model based method was proposed as a solution for this problem by detecting if the differences in the sequence came from recombination or mutation events [14]. The model, implemented in the ClonalFrame approach, relies on the analysis of the sequence of each locus and assumes that recombination occurs only with alleles not represented in the population, an unlikely assumption for many bacterial species that have been extensively sampled. The resulting distance matrix can then be represented as an UPGMA tree with bootstrap support or as a graph that should describe the contributions of both recombination and mutation.

Nevertheless, the most popular analysis of the allelic profiles in order to infer an hypothetical phylogenetic relationship between STs is that performed by the eBURST algorithm [8]. This algorithm allows for an unrooted tree-based representation of the relationship of the isolates analyzed, based on the number of differences in the allelic profile, assigning isolates to clonal complexes (CCs). The main advantage of eBURST is that it implements the simplest model for the emergence of clonal complexes [15,16]: a given genotype increases in frequency in the population, as a consequence of a fitness advantage or of random genetic drift, becoming a founder clone in the population, and this increase is accompanied by a gradual diversification of that genotype, by mutation and recombination, forming a cluster of phylogenetically closely related strains. This diversification of the "founding" genotype is reflected in the appearance of STs differing only in one housekeeping gene sequence from the founder genotype – single locus variants (SLVs). Further diversification of those SLVs will result in the appearance of variations of the original genotype with more than one difference in the allelic profile: double locus variants (DLVs), triple locus variants (TLVs), and so on. Upon application of the eBURST algorithm to an entire data set, the result is a forest, a disjoint set of trees (acyclic graphs), where each tree corresponds to a clonal complex. Those trees are the result of the application of a set of rules based on the model just described to the graph representing all SLV links. Thus, by considering only SLV links, eBURST does not aim at linking the entire population but identifies different clonal complexes.

The final eBURST forest provides us with an hypothetical pattern of descent for the strains analyzed, illustrating the possible phylogenetic relationships between STs [8,17]. The reliance on the comparison of allelic profiles buffers eBURST against the possibility of the introduction of multiple sequence changes by a single recombination event.

Another current data analysis methodology for sequence based typing techniques relying on the difference between two allelic profiles such as MLST and Multilocus Variable Number of Tandem Repeats Analysis (MLVA), consists of computing a Minimum Spanning Tree (MST) [18-21]. An MST is a tree that connects all entries in such a way that the summed distance of all links of the tree is the shortest (minimum) [22-24]. In a biological context, the MST principle and a maximum parsimony principle share the idea that evolution should be explained with as few events as possible. The main difference between the two is that parsimony methods allow the introduction of hypothetical samples, that are created to construct the internal nodes of the tree, whereas the real samples from the data set are represented as the leaves of the tree. Those hypothetical samples, are assumed to be common ancestors of the current population that can no longer be sampled. In the Bionumerics(tm) software MLST data can be analyzed has a minimum spanning tree using a simple categorical coefficient, and the algorithm was adapted to include hypothetical STs whenever such an assumption decreases significantly the total span of the tree. These hypothetical STs usually correspond to missing types for which a number of SLVs are present in the data set [25] and that are assumed to exist but not to have been included due to incomplete sampling. Although this is a reasonable assumption with the model of clonal expansion and diversification described above, it is a clear departure from the MST problem, since one postulates the existence of nodes that were not represented in the sampling and that clearly affect the MST topology. A tool available at the Pubmlst website [3] also allows users to construct MSTs based on MLST data, but on the same page a link to a tool allowing a BURST analysis of the data is also offered and the relationship between the two methods is unclear. Up to now, MSTs and eBURST for the analysis of MLST data have been considered distinct methodologies.

In spite of both eBURST and the MST approach implemented in Bionumerics(tm) being clearly related to the MST problem, they present some particularities that deserve clarification. The main difference between the two methodologies is that MST will always connect all the STs without forming defined clonal complexes as eBURST does. Using MSTs, those clonal complexes have to be infered by using empirical rules based on other data available for the isolates, or by a pre-defined allelic distance. If the later is chosen, the most common definition of CC is a group of STs that have at least six alleles in common with another member of the defined group. In the MST problem one deals with a weighted graph for which one searches for a spanning tree whose sum of link weights is minimum. However, when analyzing MLST data the methods do not clearly define link weights or have identical weights for most of the links. The simple categorical coefficient of Bionumerics(tm) uses the number of allelic differences between STs, similarly to the eBURST approach. In a situation where multiple links connecting different ST nodes have identical weights a number of solutions to the MST problem exist. To overcome the degeneracy of the MST problem and present a unique solution, these methods usually consider a quality ordering of the links relying on additional measures of profile similarity. Bionumerics(tm) also has a feature to include sets of rules based on BURST rules (using only the number of SLVs and DLVs) or to add other user defined rules. In order to present the unique solution, the combinatorial optimization problem becomes finding a tree with the highest possible quality. The MST problem and the Maximum Weight Forest problem are particular cases of graphic matroids [26]. Thus, finding a solution to represent MLST data by the use of MSTs consists of solving instances of graphic matroids [26-28] which can be optimally solved with a greedy approach [29]. We should also note that many optimal solutions for a given instance may exist that have the same quality. Formally, this happens when considering the quality sorted lists of links for two different optimal trees, the link lists have the same size and, for each list position, links in both lists have the same quality [24,26].

The eBURST algorithmic implementation can be enunciated as follows: the STs are clustered with respect to their number of SLVs, DLVs, TLVs and occurrence frequency. Given a graph where each ST is a node and where a link between two STs exists whenever they are SLVs, we want to construct a forest, i.e. a collection of disjoint trees, such that each ST connects to the neighbor with highest number of SLVs. In case of a tie, we should consider the number of DLVs, followed by TLVs and lastly by the neighbors occurrence frequency.

In the current eBURST implementation [8,17] a two step approach is used for each disjoint graph. In the first step, the algorithm builds a tree by doing a breadth-first search (BFS) starting from the group founders, i.e. the STs with highest rank in the disjoint graphs, considering the tiebreak rules of number of SLVs, DLVs, TLVs and occurrence frequency. The BFS is done iteratively. First, the group founder is connected to all its SLVs, then, each one of those, following the ST ordering, is connected to their SLVs not yet present in the tree. This process is repeated until every element of the disjoint graph is present in the tree. The second step consists in a local optimization of the tree. For each ST on the tree, the algorithm checks if a SLV exists with higher rank than the current parent. If such SLV exists, and if it is not a grandparent of the current ST, the link is deleted and the higher rank SLV becomes the new parent of the ST, thus locally improving the tree. The grandparent check is needed to verify that the tree is not broken nor does it form loops. As we have mentioned, the initial BFS tree is locally optimized, therefore, the obtained solution may not be the optimal solution. Nevertheless, and although eBURST v3 readme file [17] suggests that the problem cannot be solved without using ad-hoc rules, the described problem can be stated as a graphic matroid [26,28]. These problems can be optimally solved with the algorithm of Kruskal [23,24,26]. Thus, in this article we provide the formulation of this problem and we solve it with the mentioned algorithm (goeBURST – global optimal eBURST). We also provide an exhaustive analysis of the existing biological data sets  and comparison of the results with both implementations. To allow readers to use the new implementation, a prototype software is provided at  where users can analyze their data sets with the proposed algorithm.

## Results and discussion

### Algorithm

In this section we provide a formal definition of the problem to be solved and the proposed algorithm. The eBURST analysis of MLST results consists of building a spanning forest in a graph where each ST is a node and two STs are connected if and only if they are SLVs. Since this forest should be optimal with respect to link selection, we want to select links between STs with higher number of SLVs. In case of tie we should consider the number of DLVs, the number of TLVs and lastly the occurrence frequency of STs. Thus, given a graph *G *and the set ℱ of all forests over *G*, i.e., a matroid [26-28], the optimization problem is to find an optimal forest. As we mentioned before, the current implementation of the BURST rules in the eBURST algorithm may not provide an optimal solution. In order to achieve this goal we propose the following algorithm: if we start with a forest of singleton trees (each ST is a tree), we can build the optimal forest by iteratively selecting links connecting STs in different trees and with the higher number of SLVs. In the current implementation of the eBURST algorithm, it is implicitly defined a total order for links based on the number of SLVs, DLVs, TLVs and occurrence frequency of the connected nodes. This set of rules is what we defined as eBURST rules, i.e., the BURST rules as implemented in the eBURST software. In the proposed algorithm we include as last tiebreak rule the assigned ST number (ID). Although this last tiebreak is rarely reached, this criterion is necessary to provide a consistent and unique solution to the problem as, independently of the sorting algorithm used, it will always provide a consistent tiebreak solution due to the uniqueness of ST ID. As implemented, lower ST IDs take precedence over higher ST IDs. The rational for this choice was that, assuming a growing database with data of several contributing international studies, the more common STs are sampled first and will have lower ST IDs than the subsequent studies that will add more STs do the database.

#### Problem formalization

Let  be the set of STs and let  be a function such that *λ*(*u*, *v*) is the number of variable loci between STs *u *and *v*, i.e.,

(1)

where *π*(*u*) is the allelic profile of *u *and *δ *is the Kronecker delta function.

Given *G *= (*V*, *E*), where *V *=  and *E *= {(*u*, *v*) ∈ *V*^2^|*λ*(*u*, *v*) = 1}, we define a total order ≤ on *E *by comparing the number of SLVs, DLVs, TLVs and the occurrence frequency of the nodes. Let *μ*:  → N^4 ^be a function such that *μ*(*u*) is a vector which components are the numbers of SLVs, DLVs, TLVs and the occurrence frequency of *u *∈ . Given that these values should be relative to the connected component (clonal complex) *C *containing *u*, we formally define *μ *as

(2)

for *i *= 1, 2, 3, and *μ*(*u*)_4 _is simply the occurrence frequency of *u*. Then, given (*u*_1_, *v*_1_), (*u*_2_, *v*_2_) ∈ *E*, we say that (*u*_1_, *v*_1_) ≤ (*u*_2_, *v*_2_) if and only if *ε *≥ 0, being *ε *computed as follows, starting with *i *= 1:

1. set *ε *← max {*μ*(*u*_2_)*i*, *μ*(*v*_2_)_*i*_} - max {*μ*(*u*_1_)_*i*_, *μ*(*v*_1_)_*i*_};

2. if *ε *= 0, then set *ε *← min {*μ*(*u*_2_)_*i*_, *μ*(*v*_2_)_*i*_} - min {*μ*(*u*_1_)_*i*_, *μ*(*v*_1_)_*i*_};

3. if *ε *= 0 and *i *< 4, then set *i *← *i *+ 1 and go to step 1, otherwise return *ε*.

To illustrate the problem with a practical example consider links (211, 300) and (56, 99) from the largest connected component/clonal complex in *Burkholderia pseudomallei *data set. We have



and, by computing *e*, we know that (56, 99) ≤ (211, 300) with *ε *= 11 computed at DLV level. Note that the connected component *C *is unequivocally identified for each *u *∈ .

#### eBURST algorithm

In this section we detail the current eBURST implementation for comparison purposes. eBURST algorithm implementation performs two major steps, namely an expansion step and a local optimization step. In the first step the algorithm expands a tree from the ST with the highest number of SLVs as follows:

1. select a ST with maximum number of SLVs (in case of tie select by DLVs, TLVs and finally by ST frequency), add it to list *L*_0 _and set *d *← 0;

2. sort *L*_*d *_in decreasing order of SLVs (in case of tie consider DLVs, TLVs and at last ST frequency);

3. for *u *∈ *L*_*d*_, add all new discovered SLVs of *u *to *L*_*d*+1 _as descendents of *u*, i.e., add SLVs of *u *not yet in the tree;

4. if *L*_*d*+1 _≠ ∅, set *d *← *d *+ 1 and go to step 2.

Note this expansion may not get an optimal solution, thus the algorithm runs a second step where it tries to optimize the tree. In this second step we should consider each *L*_*d*_, for *d *≥ 2, and proceed as follows:

1. for each *u *∈ *L*_*d *_select the best SLV *v *of *u*;

2. if *v *is not a descendent of *u *in the expansion step, *v *becomes the parent of *u*.

This last condition avoids broken trees resulting from local optimization. Note also that, whenever we set the parent for a given node, it implies adding a link to the spanning tree.

The above algorithm should be executed for each connected component, i.e., we obtain a spanning forest. And, with respect to the space and time complexities, it requires *O*(*V *+ *E*) space to store the graph and it takes *O*(*V *log *V *+ *E*) time, note that expanding all adjacencies takes *O*(*E*) time and sorting the lists takes *O*(*V *log *V*) time. Since building the graph takes *O*(*V*^2^) time, the running time is dominated by graph construction.

#### goeBURST algorithm

The problem that we want to solve may be formalized as follows: find a list *L *of links that is maximized with respect to ≤, i.e., given other list *L' *and assuming that both lists are sorted against ≤, *L *is such that *L'*[*i*] ≤ *L*[*i*], for *i *= 1,..., |*L*|. Note that any list with links of a spanning forest of *G *must have the same size. This problem can be solved by a family of well known greedy algorithms [23,29].

As mentioned above, the algorithm can be stated as finding the maximum weight forest or, depending on weight definition, as finding the minimum spanning tree. All these problems are particular cases of a general class of problems known as matroids, namely graphic matroids. The Kruskal algorithm [23,24,26] provides an optimal solution for this problem class and, given *G *= (*V*, *E*), it works as follows:

1. sort *E *with respect to the total order ≤ in decreasing order;

2. create a forest *F *where each *u *∈ *V *is a tree;

3. iterate over *E *in decreasing order and, for each (*u*, *v*) ∈ *E*, if *u *and *v *are in different trees, add (*u*, *v*) to *F *combining both trees as single tree;

4. return *F*.

The goeBURST algorithm takes *O*(*V *+ *E*) space to store the nodes, links and all necessary information. It takes *O*(*E *log *V*) time to sort the links, *O*(*V*) time to create the initial forest and *O*(*Eα*(*V*, *E*)) to build the optimal forest, where *α *is the well known inverse of Ackermann function. The last complexity term arises from the use of disjoint sets data structure to efficiently trace the nodes in each tree [24]. Since building the graph takes *O*(*V*^2^) time and computing the connected components and the vectors *μ *for all STs takes *O*(*V *+ *E*), the running time is dominated by the graph construction. Thus, analyzing a MLST instance takes *O*(*V*^2^) time.

#### Groups defined at higher allelic distances

As described in the previous section, the resulting forest (where each tree is a clonal complex) is defined for an allelic distance of one, i.e, the minimum distance of any ST to at least one of the STs of the same Clonal Complex is a difference in a single locus. The user may choose to display sets of isolates related at higher levels than SLV but those are represented as sets of disjoint CCs, i.e., as a forest of trees each one connected at SLV level. Nevertheless, groups can also be defined at distances greater than one allele difference, iteratively following the same set of eBURST rules for link selection. As an example, to define groups at TLV level, the rules are first applied to the selection of all SLV links between the STs, and afterwards all the possible links between CCs or STs that are DLVs are evaluated using the same rules, and the best DLV link for each pair of CCs/STs is selected. The same process is then applied to STs or groups that can have TLV links between them.

In the current eBURST implementation, the connection between STs at levels higher than SLV is not explicitely represented. In the goeBURST implementation, the user has the choice to link these forests to create a single tree for each clonal complex, up to TLV level. As stated above, the same eBURST rules can be followed to choose the single link to be represented connecting disjoint CCs or STs. The choice of the TLV level as the upper level of connection to be displayed was based on two factors: 1) the defined rules for eBURST only use tiebreaks up to TLV level, so creating groups higher than TLV will not make any changes in the drawn links and would only further join CCs/STs, and 2) the number of links to be evaluated grows quadratically with the number of STs, being computationally intensive in terms of time and memory for large data sets. An important feature in the goeBURST algorithm is that, similarly to the eBURST implementation, the number of DLVs and TLVs is always calculated for each group defined at the chosen allelic distance. This means that, for the purpose of link selection, only DLVs and TLVs that are within that group are taken into account. Therefore higher allelic distances may result in a higher number of DLVs or TLVs for each ST that can influence the decisions reached by the tiebreak rules and which links are effectively drawn.

Examples of population snapshots created with groups defined a TLV level for *Staphylococcus aureus *[See Additional File 1] and *Streptococcus pneumoniae *[See Additional File 2].

### Testing

goeBURST was applied to several public MLST databases for clinically relevant microorganisms: *Helicobacter pylori*, *Streptococcus pyogenes*, *Bacillus cereus*, *Streptococcus pneumoniae*, *Pseudomonas aeruginosa*, *Enterococcus faecalis*, *Klebsiella pneumoniae*, *Campylobacter upsaliensis*, *Streptococcus suis*, *Neisseria spp.*, *Haemophilus influenzae*, *Campylobacter jejuni*, *Streptococcus uberis*, *Staphylococcus epidermidis*, *Staphylococcus aureus*, *Streptococcus agalactiae*, *Burkholderia pseudomallei *and *Enterococcus faecium*. These databases were retrieved from public MLST database repositories available in different websites (see Acknowledgments for more details). In order to provide examples of the results for different species obtained by the goeBURST algorithm, population snapshots were created for the following species: *Staphylococcus aureus *[see Additional Files 1 and 3], *Streptococcus pneumoniae *[see Additional Files 1 and 4], *Neisseria spp. *[see Additional File 5], *Enterococcus faecium *[see Additional File 6] and *Burkholderia pseudomallei *[see Additional File 7].

Of those allelic profile databases tested, five presented differences between the current locally optimized implementation of the eBURST rules and goeBURST. These results are presented in Table 1. The differences observed are due to the heuristic optimization procedure of the eBURST implementation described in the Introduction, since the local optimization fails to consider all the possible ties at a certain tiebreak rule. In contrast, goeBURST will always provide an optimal solution for the link assignment, since it performs a global optimization taking into consideration all possible ties at all levels between STs in the data set.

**Table 1:** Comparison between eBURST and the proposed implementation.

	# diffs	Group		Ties		Links	
Dataset		founder	size	#	breaker	created	deleted
*Enterococcus faecium*	1	17	268	3	SLV	50–177	50–204

*Streptococcus*	3	124	78	3	SLV	392–2803	440–2803
*pneumoniae*		138	189	14	SLV	171–361	338–3163
		217	33	2	SLV	618–3577	1325–1331

*Burkholderia*	4	48	348	3	SLV	435–667	116–435
*pseudomallei*		48	348	53	DLV	211–300	56–99
		48	348	3	SLV	70–290	66–67
		48	348	4	DLV	24–643	570–643

*Campylobacter*	5	21	849	29	SLV	104–492	474–577
*jejuni*		21	849	14	SLV	353–462	2395–2517
		21	849	2	SLV	2141–2842	1076–2951
		21	849	4	SLV	824–2141	878–2141
		177	53	2	SLV	1022–1503	1387–2162

*Neisseria*	7	11	296	18	SLV	8–1058	66–1058
*spp.*		11	296	1	SLV	10–2174	10–5091
		22	344	23	SLV	23–1062	1062–1625
		60	164	9	SLV	1157–1421	1421–1649
		269	322	21	SLV	275–352	352–1163
		1583	67	1	SLV	1905–6717	1579–1901
		1583	67	4	SLV	1590–1928	1599–1903

The following data sets presented no differences: *Helicobacter pylori, Streptococcus pyogenes, Staphylococcus aureus, Bacillus cereus, Pseudomonas aeruginosa, Enterococcus faecalis, Klebsiella pneumoniae, Campylobacter upsaliensis, Streptococcus suis, Haemophilus influenzae, Streptococcus uberis, Staphylococcus epidermidis *and *Streptococcus agalactiae*.

**Figure 1:**
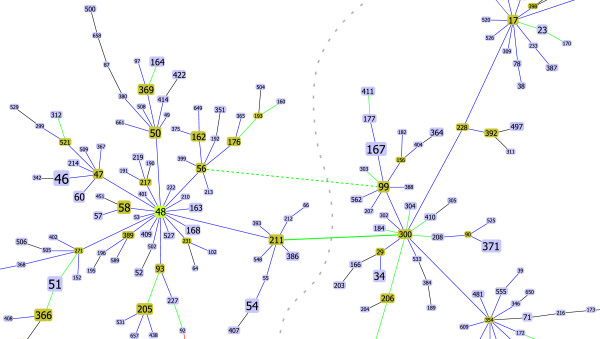
**Partial snapshot of Burkholderia pseudomallei CC 48**. Partial snapshot of *Burkholderia pseudomallei *CC 48 highlighting one of the differences (bold green lines) between current eBURST implementation (dashed lines) (ST 99 – ST56) and the proposed algorithm (full lines)(ST211 – ST300). The gray interrupted line represents the decision in the tiebreak that needs to be taken. The tiebreak level was at DLV level indicated by the green color of the link. See text for further details.

**Figure 2:**
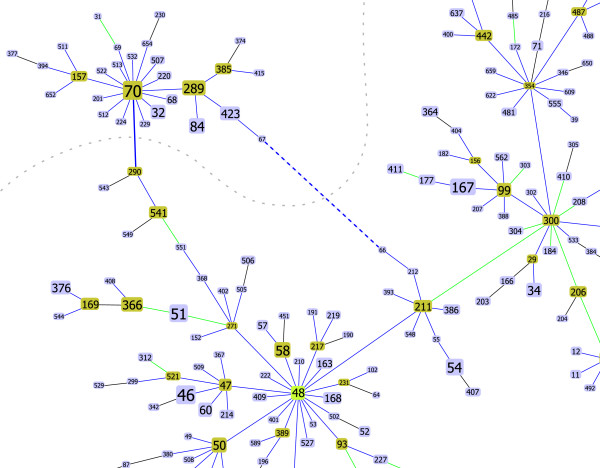
**Partial snapshot of Burkholderia pseudomallei CC 48**. Partial snapshot of *Burkholderia pseudomallei *CC 48 highlighting one of the differences (bold blue lines) between current eBURST implementation (dashed lines)(ST66–ST67) and the proposed algorithm (full lines)(ST70–ST290). The gray interrupted line represents the decision in the tiebreak that needs to be taken. The tiebreak level for this example was at SLV indicated by the blue color of the link. See text for further details.

An illustration of the type of differences that can be observed in the patterns of descent for *Burkholderia pseudomallei *can be seen on Figure 1 and Figure 2. In Figure 1 a partial view of the CC48 (clonal complex with ST48 as founder) is shown, highlighting the link between ST211 and ST300. This link represents the assignment that conforms to all rules, from 53 possible options (ties) when creating the tree using eBURST rules. The current eBURST implementation returns the pair ST56–ST99 as the drawn link. Although both possible pairs have the same relationship in terms of number of SLVs (both ST300 and ST99 have 13 SLVs while ST211 and ST56 have 12 SLVs), the link between ST300 and ST211 should be drawn according to the eBURST rules, since the STs in this pair have more DLVs (ST300 has 57 DLVs and ST211 has 50 DLVs while ST56 has 46 DLVs and ST99 has 36 DLVs). This change completely alters the proposed pattern of descent within CC48, with ST211 and closely related STs constituting a bridge between the group founder (ST48) and closely related STs with a marked star-like topology and a more dispersed set of STs with a more linear topology. Figure 2 illustrates another change that occurs within CC48. The representation of a link between ST70 and ST290 instead of the link between ST66 and ST67 provides a better solution for a simpler tiebreak with only 3 ties at the SLV level.

#### Link confidence assessment

Another advantage of having a global optimal solution is the possibility of using the distinctiveness of a link among all possible links that could have been drawn as a confidence measure for the drawn link. The measure is based on the level of the tiebreak needed to decide if a certain link should be selected in detriment of others. The higher the level of tiebreak rule reached before such a decision can be made, the smaller the difference between similar links exist in the list of ties at that level and less confidence we have on link selection. This is a direct result of the tiebreak rule order of eBURST implemented in goeBURST.

The higher the level of tiebreak rule invoked to support the decision, the more probable it is that the identification of new STs, by more extensive sampling of the population, will change the number of SLVs, DLVs or TLVs, within the CC under study leading to the replacement of these lower quality links. It is important to note that to ensure uniqueness of the solution, a final and decisive tiebreak based on the ST identification number was implemented as previously described in the Algorithm subsection.

In Figures 1 and 2 examples using the *Burkholderia pseudomallei *data set are represented. Links where the tiebreak occurred at the number of SLVs, DLVs and TLVs are shown by the goeBURST software in blue, green and red lines respectively. Black lines are links where no tie was found.

#### Changes in the studied data sets

##### Enterococcus faecium

**Figure 3:**
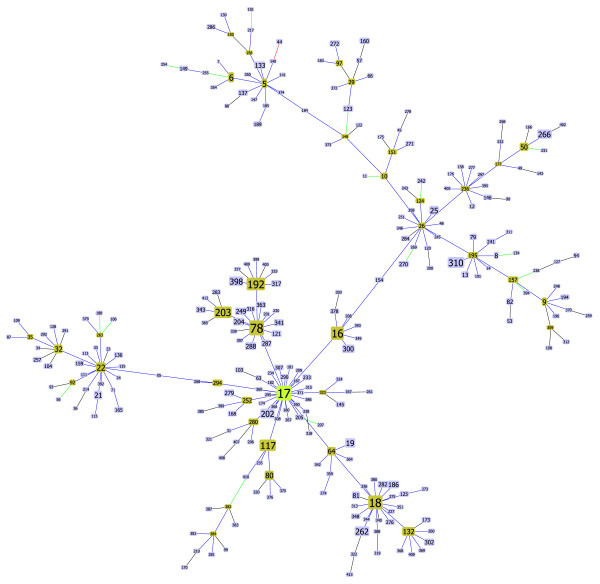
**Clonal Complex 17 of Enterococcus faecium**. Representation of the largest clonal complex for *Enterococcus faecium*, with ST17 as the determined founder.

Only one difference was found in the *Enterococcus faecium *data set studied. The changed link occurs in the largest eBURST clonal complex for the data set (containing 65% of the STs in the data set and 84% of the isolates), with ST17 as the assigned founder (Figure 3). Recent molecular epidemiology studies of *E. faecium *have shown the existence of a genetic lineage, within this largest clonal complex designated "CC17" [30-32]. This designation refers only to a subgroup of STs within the largest clonal complex defined by eBURST, that are associated with hospital outbreaks and isolates recovered from infections in hospitalized patients. The importance of the detected incorrect link between ST50 and ST204, lies in the fact that ST204 is part of "CC17" while ST177 is not. The new link connects ST50 with ST177, outside the "CC17" group, which could lead to the redefinition of "CC17" patterns of descent.

##### Streptococcus pneumoniae

The differences described in Table 1 occur in 3 different clonal complexes: CC124, CC138 and CC217. For CC124 and CC138, the observed changes alter the pattern of descent within each CC. In CC124 the sub-group founder ST392 has now a shorter SLV path to the group founder ST124 than the sub-group founder ST440. The other affected group, CC138 is the second largest group in the *Streptococcus pneumoniae *data set with 189 STs and 380 isolates (approximately 5% of the entire data set for both number of STs and number of isolates). The observed change completely altered the pattern of descent from CC176, the largest sub-group founder and possibly the most plausible group founder since it has only 2 SLVs less than ST138 but has more DLVs and TLVs and a more marked star-like topology. The change observed in CC217 is more subtle involving the evolutionary path between the founder ST and the hypervirulent ST618 [18], that is the ST representative of more isolates in the CC.

##### Burkholderia pseudomallei

**Figure 4:**
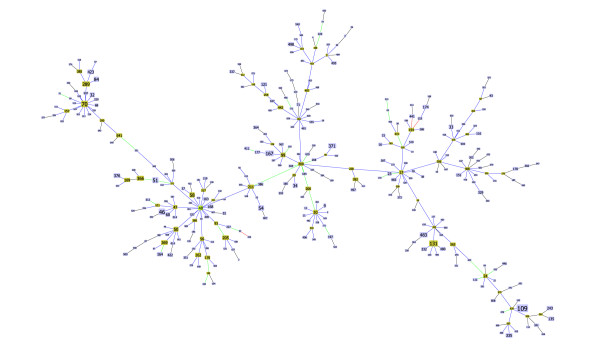
**Clonal Complex 48 of Burkholderia pseudomallei**. Representation of the largest clonal complex for *Burkholderia pseudomallei*, with ST48 as the determined founder.

For *Burkholderia pseudomallei *all the differences were observed in the largest CC for the data set, with ST48 as the founder (Figure 4). This CC encompasses 53% (348 of 652) of the STs in the database and 58% (1005 of 1725) of the isolates. Two of the changes observed lead to major rearrangements in the topology of the eBURST tree (Figures 1 and 2), while the two other changes only reflect local small distance rearrangements in the tree.

Also interesting, is that a large number of links in CC48 (32 being 9.2% of the total links in the CC) were chosen at a tiebreak level of DLV and three links had a tiebreak level of TLV. This indicates that some links of this large group are unreliable and that the real pattern of descent within this group can be very different from the one produced by the optimal solution based on eBURST rules.

##### Campylobacter jejuni

In this data set, four of the five differences observed were in CC21, the largest clonal complex with 849 STs (approximately 30% of all STs in data set) and 1655 isolates (37% of the isolates in data set). Several studies identify this clonal complex as diverse and widely distributed, and associated with poultry and human isolates. Of these changes the largest impact is the creation of a link between ST2141 and ST2842 instead of a link between ST1076 and ST2951, while the other changes correspond mostly to local rearrangements.

##### Neisseria spp

The data set used contains data for *Neisseria meningitidis*, *Neisseria lactamica *and *Neisseria gonorrhoeae*, since the same MLST scheme proposed can be effectively used to type the three species. Seven differences were found between the two eBURST implementations: two in CC11 complex of *Neisseria meningiditis*, one difference each in CC60, CC20 and CC269 also from *Neisseria meningiditis *and two in CC1583 of *Neisseria gonorrhoeae*. The two changes in CC11 of *N. meningiditis *change the pattern of descent between the group founder (ST11) and the largest subgroup founder, ST8. This clonal complex has several strains of recognized clinical importance due to their invasiveness and hypothetical capsular replacement [33,34]. CC1583 of *Neisseria gonorrhoeae *is a small CC with 67 STs and 132 isolates on the data set where the changes observed drastically change the topology of the group.

### Implementation

#### Availability

The goeBURST algorithm was implemented in java and matlab (MathWorks Inc). A java interface was created allowing users to test their data sets and visualize the results of the algorithm. It is available at . It uses the Prefuse visualization toolkit [35] and the VectorGraphics package of the FreeHEP Java library [36]. For a full description of the interface capabilities a tutorial is available at  and a help section is available in the software.

#### Changing the founder of a clonal complex

One of the features in the current implementation of the eBURST algorithm is allowing the user to test an hypothesis of an alternative pattern of descent by changing the founder of a group, that eBURST defines as the ST with more SLVs. Since the current implementation relies on an heuristic local optimization procedure, when changing the assigned founder ST, the optimization could also lead to non-optimal rearrangements in the tree, leading to a solution that violates the eBURST rules. In the proposed implementation, when a new founder is selected, the algorithm draws all the links between this ST and its SLVs, effectively deciding all tiebreaks in favor of the presumed founder. This is the only observed change since the calculated tree is already optimal, however, this can still impact on the overall topology of the tree if the SLVs are themselves subgroup founders and important hubs in the tree.

## Conclusion

eBURST provides researchers with the ability to create groups of closely related strains from MLST data. A recent simulation study showed that the eBURST definition of clonal complexes and the inferred pattern of descent within them is reliable in conditions comparable to those of the majority of natural bacterial populations of many different species while also uncovering conditions when eBURST performance is suboptimal [37].

**Figure 5:**
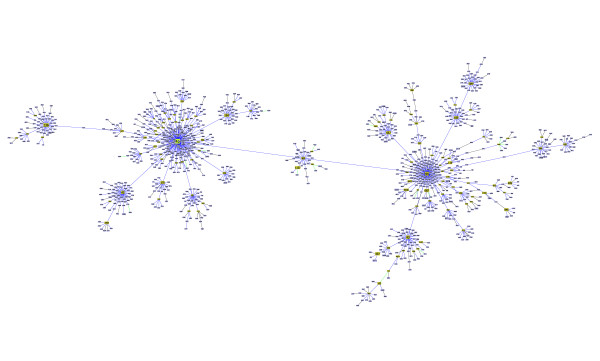
**Clonal Complex 41 of Neisseria spp**. Representation of the largest clonal complex for *Neisseria spp.*, with ST41 as the determined founder.

In this paper, we propose an algorithm for an optimal implementation of the eBURST rules. This was achieved by formalizing the problem as a matroid, namely a graphic matroid that generalizes the MST problem. Our analysis clarifies the relationship between an MST and the eBURST approach. If one considers only the allelic profile to derive an MST connecting all STs, multiple optimal solutions exist due to the limited and discrete space of ST differences. eBURST is similar to finding a MST on the entire data set but restricting the links only to those between SLVs and selecting the trees with the highest quality as defined by a set of rules. The eBURST approach uses a set of rules to create an ordered list of the links to be drawn and this optimization problem, although akin to the MST problem, should not be confused with the MST problem itself which implies a weighted graph. Formally eBURST can be stated as the more general class of combinatorial optimization problems over a matroid. This relationship between eBURST and a general problem category indicates that the optimal solution following the eBURST rules can be provided by a greedy approach identifying the optimal forest with respect to the defined partial order on the set of links between STs. To achieve that goal we propose using the Kruskal algorithm due to its desirable properties and ease of implementation, although other algorithms also provide optimal solutions to these problems. goeBURST provides a global optimal solution and corrects links that were not strictly following the eBURST rules occurring in the present software implementation, due to the use of an heuristic local optimization procedure. The changes were observed in bacteria with a high ratio of recombination to mutation, and the majority occurred in the largest clonal complex in each data set. These clonal complexes have usually a "straggly" appearance, meaning that the resulting tree diverges significantly from a star-like topology and presents a number of higher order ramifications. CC17 of* Enterococcus faecium *(Figure 3), CC41 of *Neisseria meningiditis *(Figure 5) and CC48 of *Burkholderia pseudomallei *(Figure 4) are prime examples of such straggly groups. The majority of the differences found occurred in clinically relevant clonal complexes for the bacterial species under study and significantly altered the pattern of descent within the CC, rendering them of critical importance for the epidemiological analysis based on MLST. Furthermore, the ongoing growth of MLST databases resulting from the increased sampling of bacterial populations, is expected to generate more complex optimization problems that could further bring forward the limitations of the current implementation.

MSTs have recently attracted attention for the analysis of MLST data since they share a similar principle to the maximum parsimony method frequently used in phylogenetic analysis. The Bionumerics(tm) software has been used to identify MSTs using a simple categorical coefficient and MLST data [18,20,21]. As we discussed, the analysis of MLST or MLVA data using MSTs is incompatible with a unique solution due to the limited space defining the weights of the links. The publication describing this feature of the software [19] clarifies that, with the default options, rules similar to those of BURST are used to define the quality of each link. The Bionumerics(tm) approach is therefore formally identical to the BURST implementation proposed here if one excludes the creation of hypothetical nodes and if all the rules are followed. Similarly the MST analysis using Prims's algorithm [24] provided at the Pubmlst site [3] is formally identical to the goeBURST implementation, as long as the same set of rules are used.

Considering the proposed algorithm it becomes intuitive that the pattern of descent within the clonal complexes produced as well as the clonal complexes themselves, cannot be considered static and are highly dependent on the available data. Each link has a number of ties and a level of tiebreak rule reached to decide if the link should be drawn. Through the evaluation of these two parameters one can determine if the patterns of descent within a clonal complex are robustly defined or if the availability of new data, by more complete sampling, may significantly alter the proposed evolutionary path. goeBURST allows the evaluation of link confidence through the tiebreak level reached enabling the researcher to determine by visual inspection of the tree if a drawn link represents a reliable connection between STs or if other connections with only a slightly worse quality exist, alerting for the possibility of alternative patterns of descent. At a clonal complex level, the existence of several links that were drawn at higher tiebreak levels (DLV, TLV or ST frequency) could point to two possible scenarios: insufficient or biased sampling or, alternatively, high recombination or mutation levels generating, in a very short time period, sets of allelic profiles with ties at all levels.

The general applicability of graphic matroids and the evolutionary model underpinning the eBURST rules suggests that goeBURST can be used successfully for the analysis of other microbial typing data. Any multilocus typing methodology whose results can be coded into a numeric or character sequence and in which the similarity between two such profiles can be assessed by the number of differences, is amenable to analysis by goeBURST. MLVA and CRISPRs analysis [38,39] are examples of methodologies whose results are typically analyzed by MST and where the goeBURST algorithm can be fully applied. The growing interest of SNP analysis to probe the recent evolutionary history of monomorphic bacterial pathogens [39,40] has been accompanied by the use of multiple methods to analyze the data generated. The use of MSTs in this context in different studies [40,41] without a clear reference to the tiebreak rules used, may compromise their reproducibility and comparability. goeBURST can be used in the analysis of SNP data without these caveats and using tiebreak rules based on an evolutionary model that has proven useful in the analysis of bacterial short-term evolution. Nevertheless, depending the number of loci analyzed by these methods and the distribution of the number of differences found between isolates, the tiebreak rules may need to be revised since they  were originally conceived for the analysis of seven loci MLST data.

The large body of work on graphic matroids can be tapped to provide novel analysis tools that may offer fresh insights into microbial population dynamics.

## Authors' contributions

APF designed, proposed the new algorithm, ran the tests, implemented the visualization software and wrote the paper. MB did the initial eBURST code analysis, proposed a correction and ran the tests. MR and JAC designed and coordinated the study, analyzed the results and wrote the paper. All authors read, contributed and approved the final draft.

## Supplementary Material

Additional file 1 **Population snapshot of *Staphylococcus aureus *with groups defined at TLV level**. Population snapshot of *Staphylococcus aureus *created goeBURST v1.2 software using a data set downloaded from . Gray lines define the links at DLV or TLV between the CCs (darker gray – DLV link; lighter gray – TLV links), defined following the eBURST rules (see text). Click here for file

Additional file 2 **Population snapshot of *Streptococcus pneumoniae *with groups defined at TLV level**. Population snapshot of *Streptococcus pneumoniae *created by goeBURST v1.2 software using a data set downloaded from . Gray lines define the links at DLV or TLV between the CCs (darker gray – DLV link; lighter gray – TLV links), defined following the eBURST rules (see text). Click here for file

Additional file 3 **Population snapshot of *Staphylococcus aureus *representing Clonal Complexes (defined at SLV level)**. Population snapshot of *Staphylococcus aureus *created by goeBURST v1.2 software using a data set downloaded from . Click here for file

Additional file 4 **Population snapshot of *Streptococcus pneumoniae *representing Clonal Complexes (defined at SLV level)**. Population snapshot of *Streptococcus pneumoniae *created by goeBURST v1.2 software using a data set downloaded from . Click here for file

Additional file 5 **Population snapshot of *Neisseria spp. *representing Clonal Complexes (defined at SLV level)**. Population snapshot of *Neisseria spp. *created by goeBURST v1.2 software using a data set downloaded from . Click here for file

Additional file 6 **Population snapshot of *Enterococcus faecium *representing Clonal Complexes (defined at SLV level)**. Population snapshot of *Enterococcus faecium *created by goeBURST v1.2 software using a data set downloaded from . Click here for file

Additional file 7 **Population snapshot of *Burkholderia pseudomallei *representing Clonal Complexes (defined at SLV level)**. Population snapshot of *Burkholderia pseudomallei *created by goeBURST v1.2 software using a data set downloaded from . Click here for file
