# Supplementary figures and images for: Global optimal eBURST analysis of multilocus typing data using a graphic matroid approach
Source: BMC Bioinformatics. 2009 May 18;10:152. doi: 10.1186/1471-2105-10-152 (PMC2705362; doi:10.1186/1471-2105-10-152)

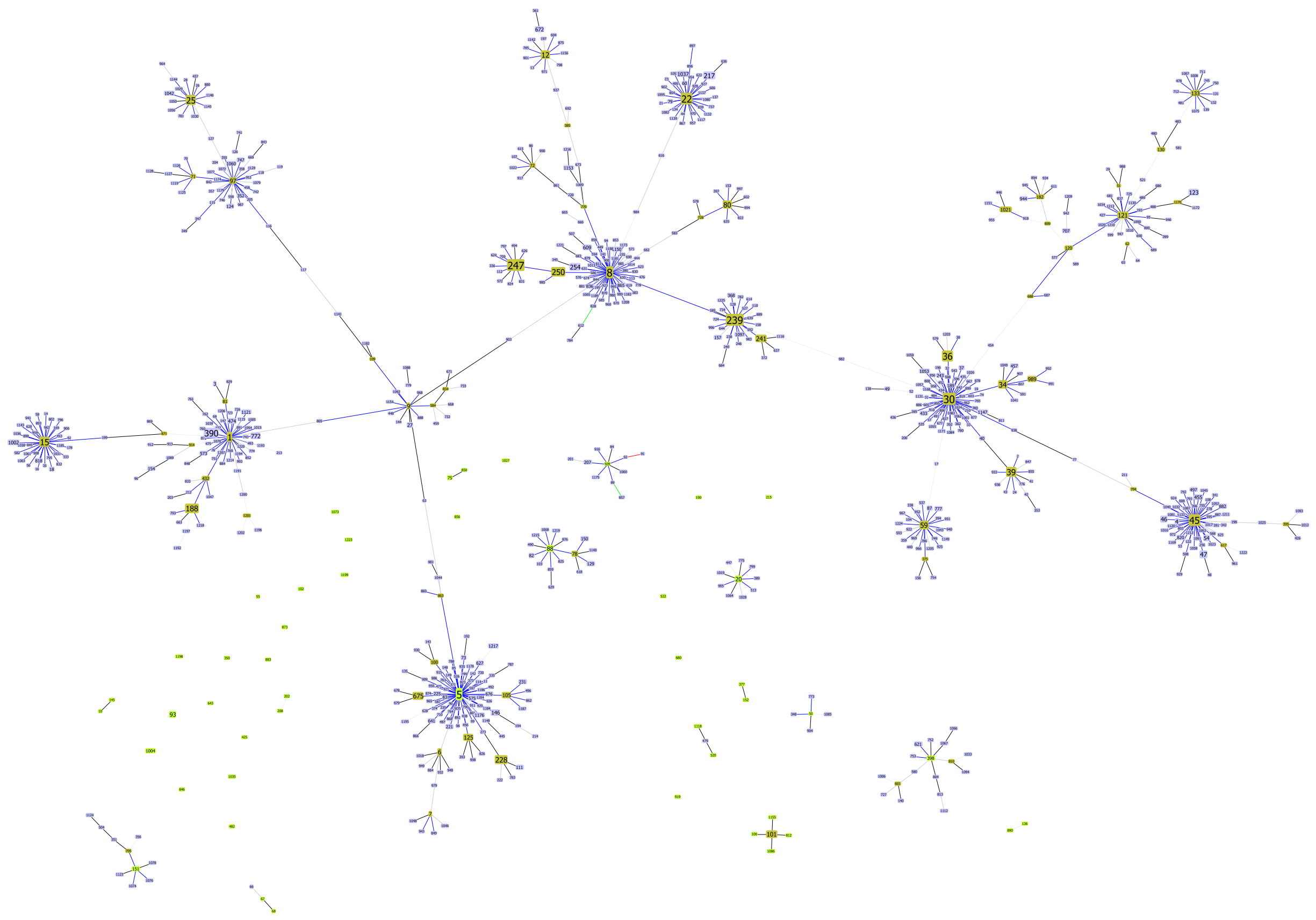

Supplement: Additional file 1 — Population snapshot of Staphylococcus aureus with groups defined at TLV level. Population snapshot of Staphylococcus aureus created goeBURST v1.2 software using a data set downloaded from . Gray lines define the links at DLV or TLV between the CCs (darker gray – DLV link; lighter gray – TLV links), defined following the eBURST rules (see text). [file 1471-2105-10-152-S1.pdf]

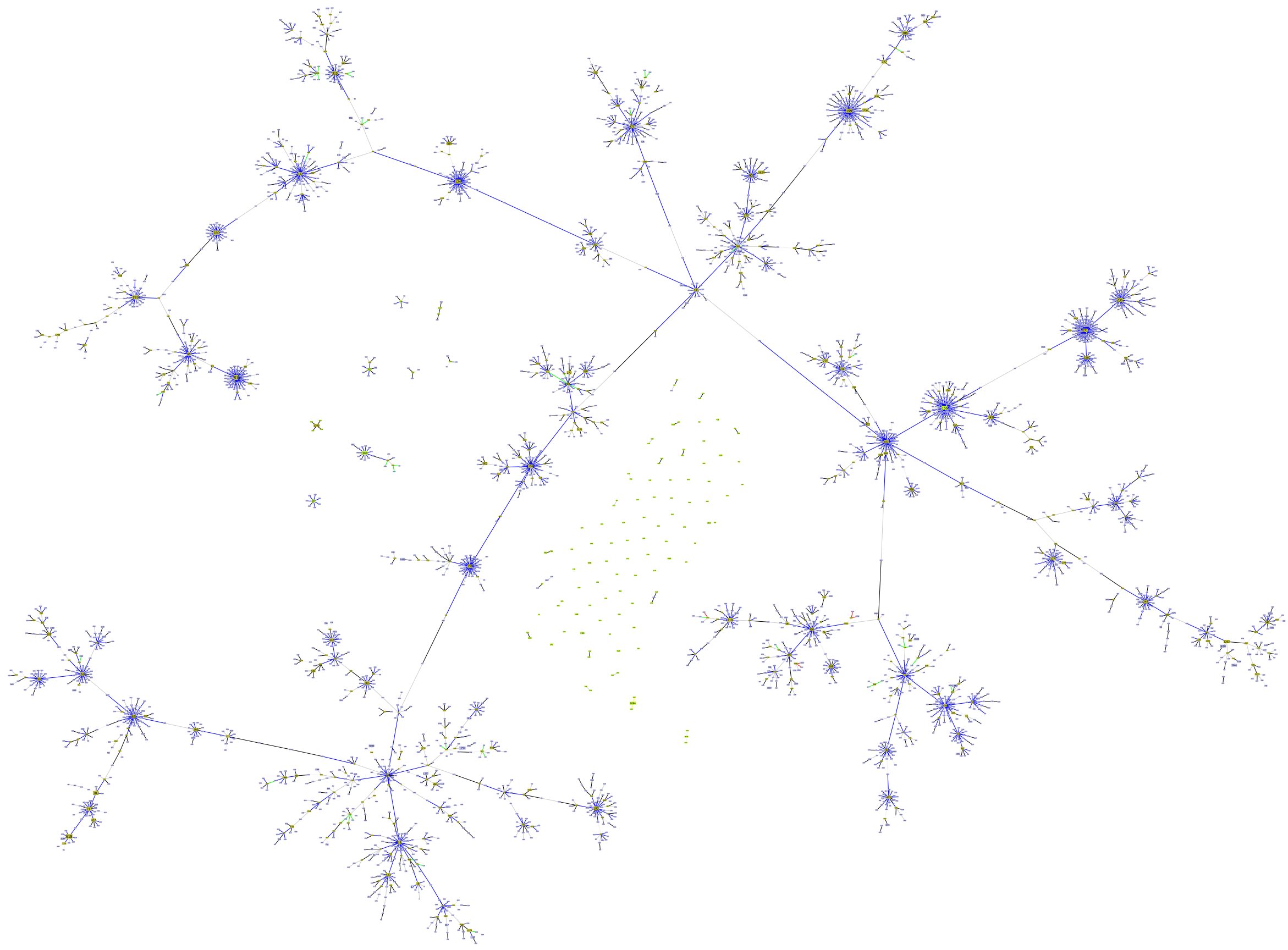

Supplement: Additional file 2 — Population snapshot of Streptococcus pneumoniae with groups defined at TLV level. Population snapshot of Streptococcus pneumoniae created by goeBURST v1.2 software using a data set downloaded from . Gray lines define the links at DLV or TLV between the CCs (darker gray – DLV link; lighter gray – TLV links), defined following the eBURST rules (see text). [file 1471-2105-10-152-S2.pdf]

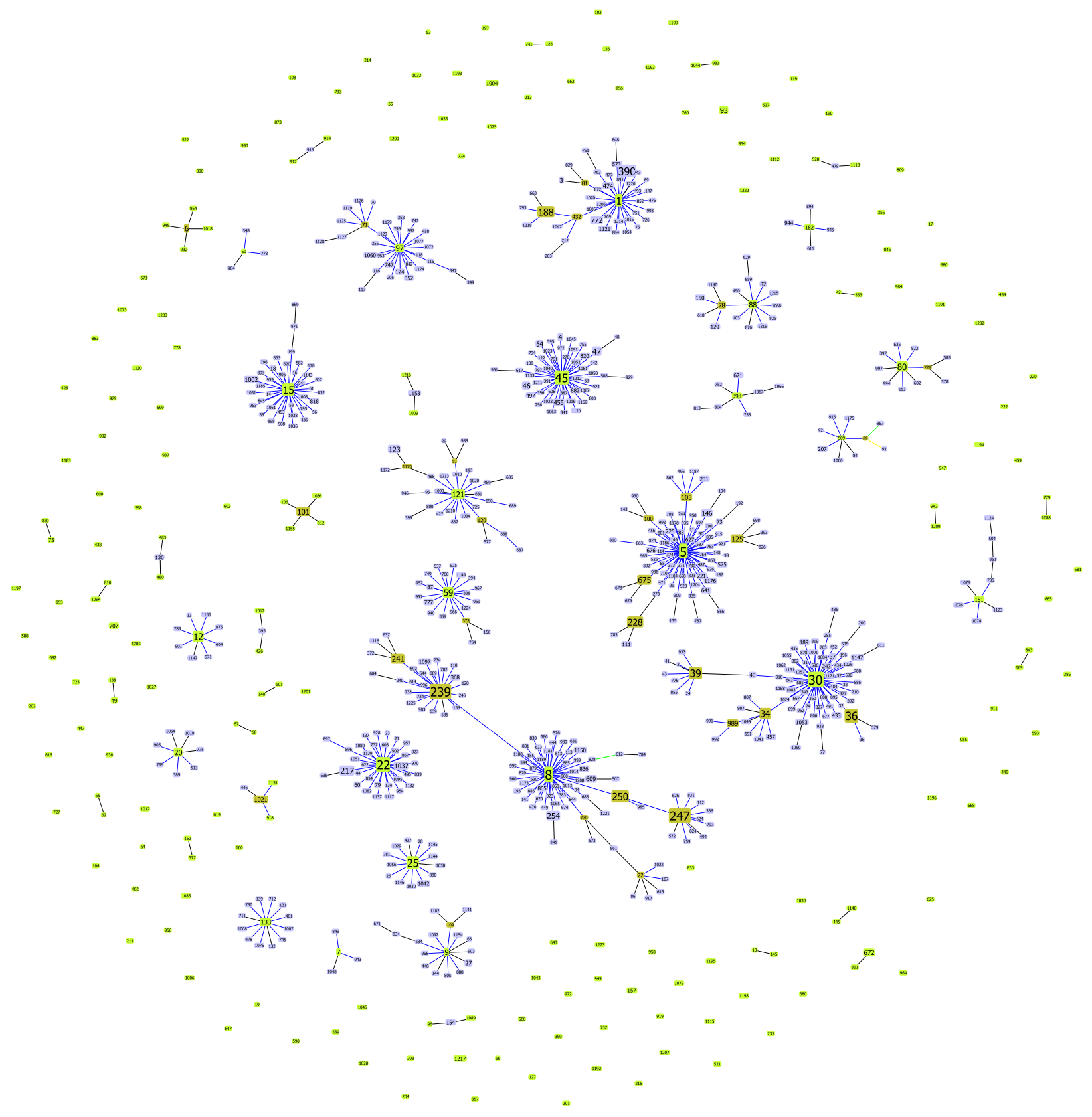

Supplement: Additional file 3 — Population snapshot of Staphylococcus aureus representing Clonal Complexes (defined at SLV level). Population snapshot of Staphylococcus aureus created by goeBURST v1.2 software using a data set downloaded from . [file 1471-2105-10-152-S3.pdf]

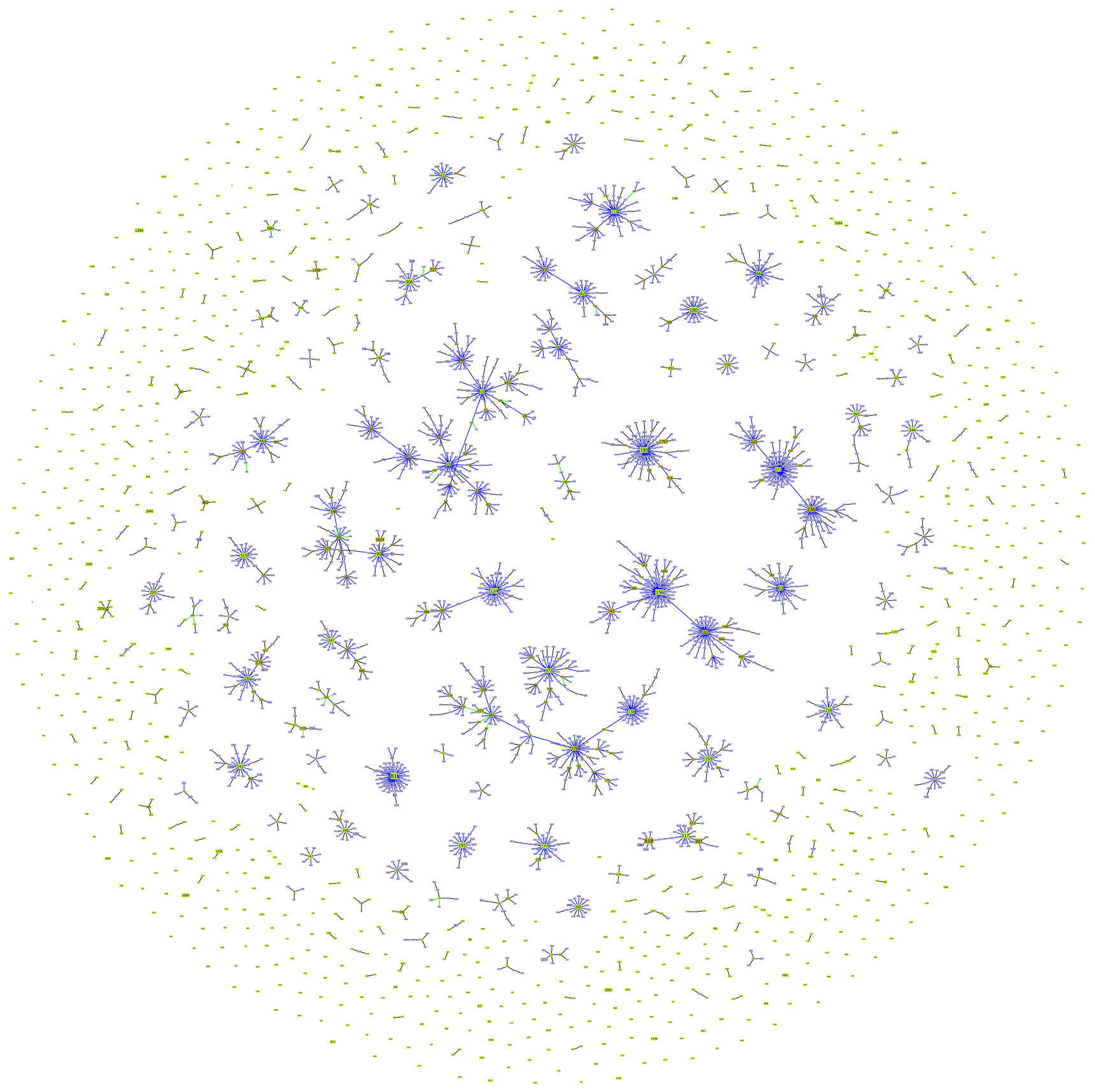

Supplement: Additional file 4 — Population snapshot of Streptococcus pneumoniae representing Clonal Complexes (defined at SLV level). Population snapshot of Streptococcus pneumoniae created by goeBURST v1.2 software using a data set downloaded from . [file 1471-2105-10-152-S4.pdf]

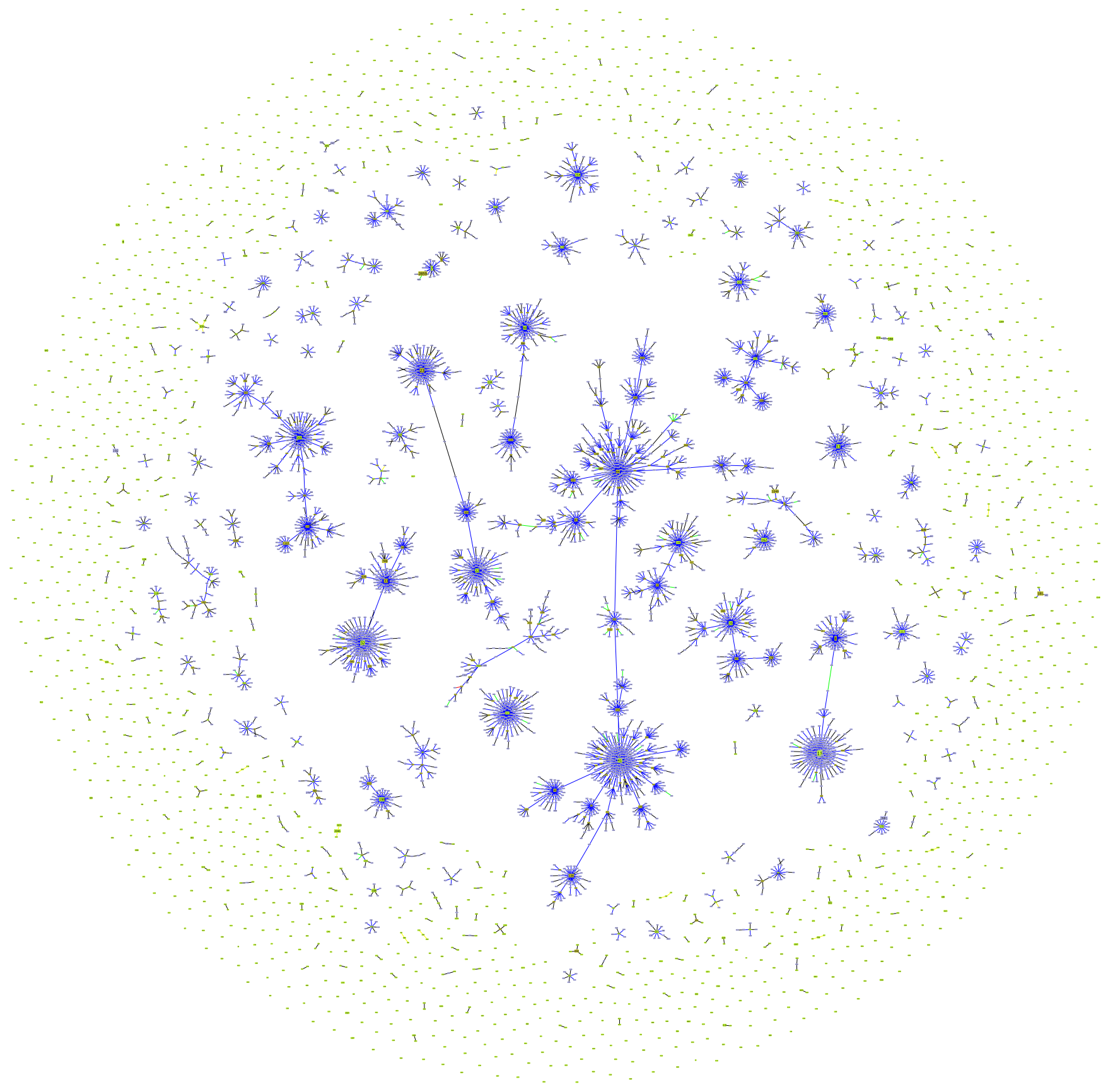

Supplement: Additional file 5 — Population snapshot of Neisseria spp. representing Clonal Complexes (defined at SLV level). Population snapshot of Neisseria spp. created by goeBURST v1.2 software using a data set downloaded from . [file 1471-2105-10-152-S5.pdf]

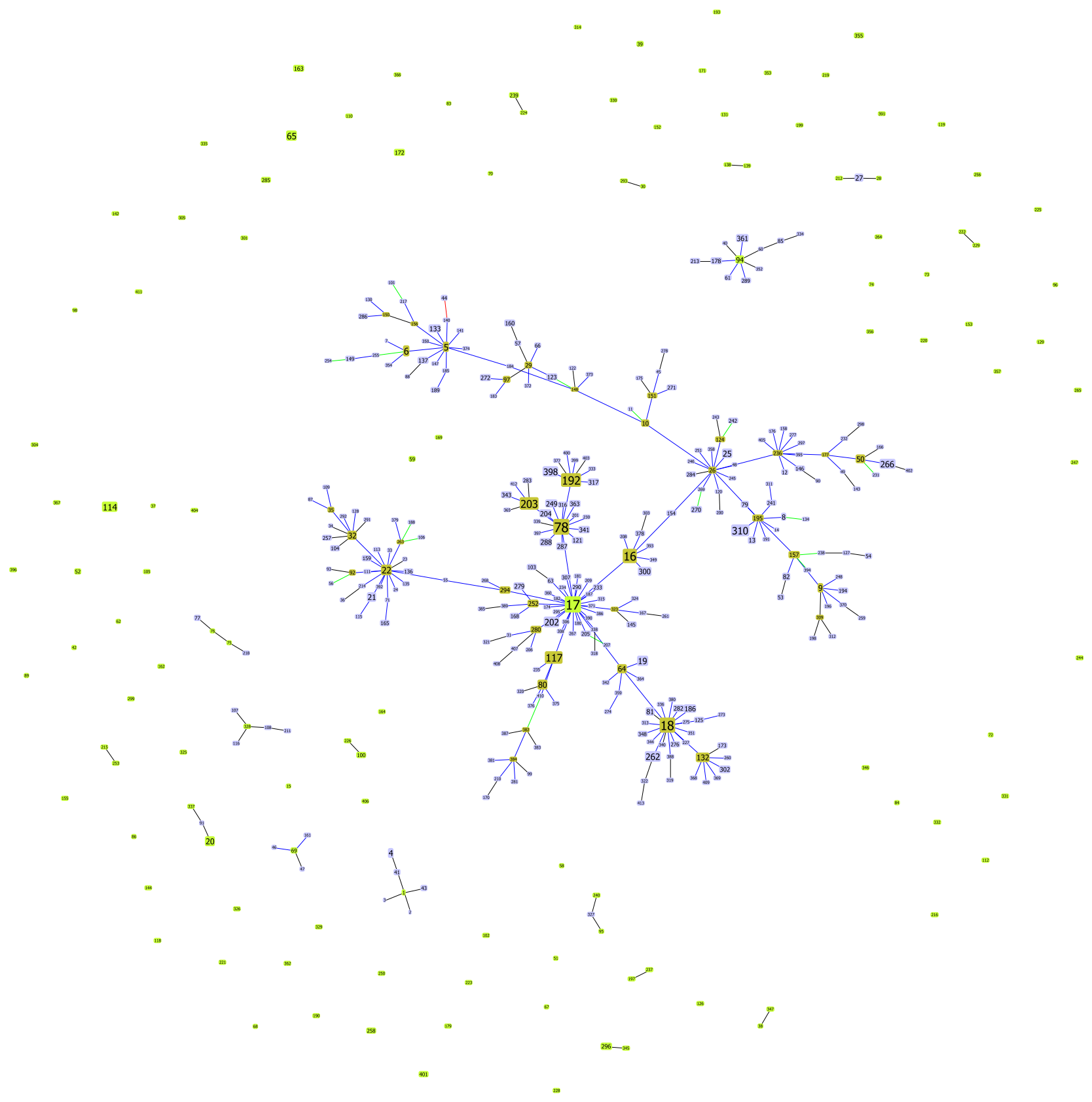

Supplement: Additional file 6 — Population snapshot of Enterococcus faecium representing Clonal Complexes (defined at SLV level). Population snapshot of Enterococcus faecium created by goeBURST v1.2 software using a data set downloaded from . [file 1471-2105-10-152-S6.pdf]

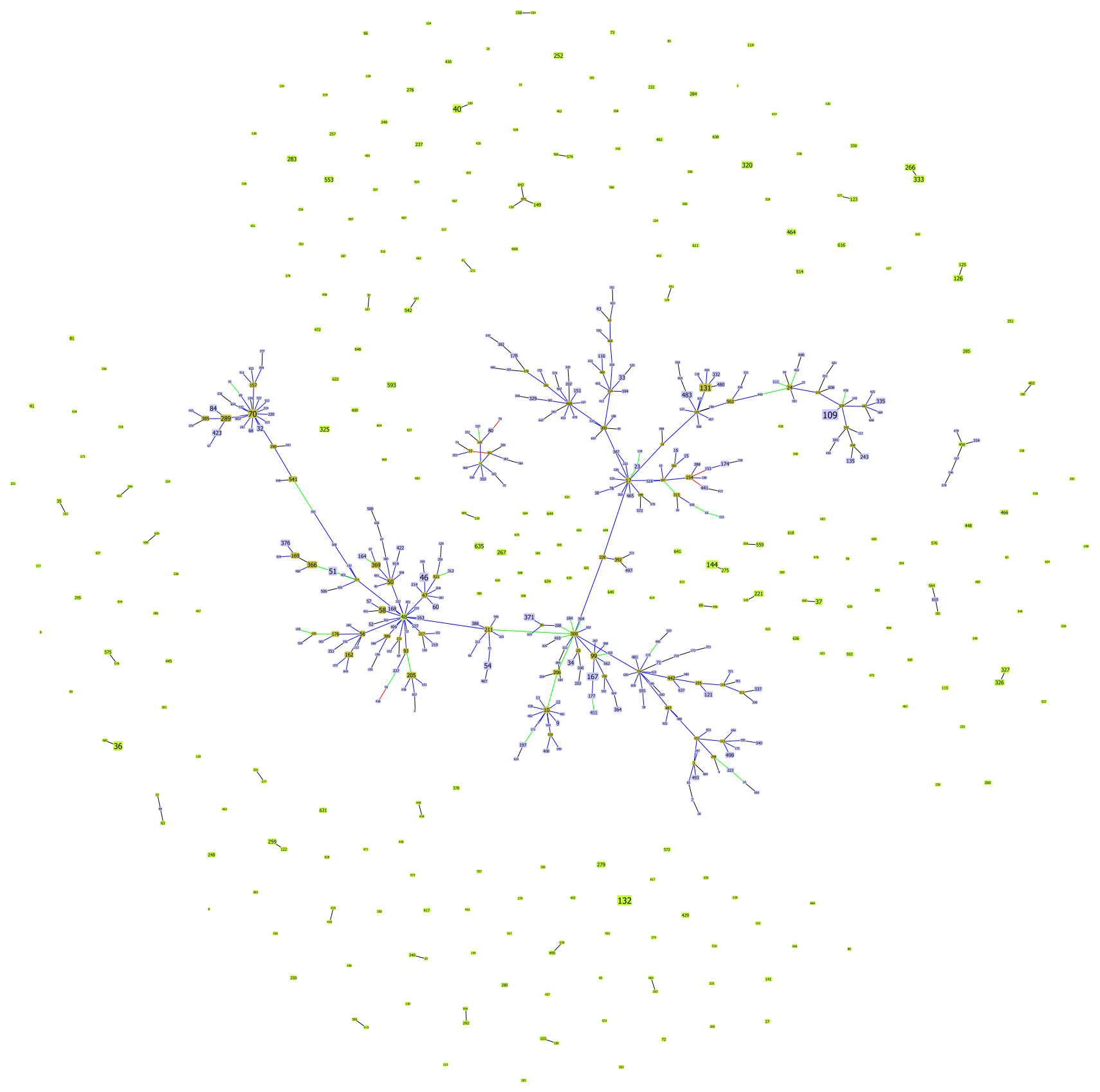

Supplement: Additional file 7 — Population snapshot of Burkholderia pseudomallei representing Clonal Complexes (defined at SLV level). Population snapshot of Burkholderia pseudomallei created by goeBURST v1.2 software using a data set downloaded from . [file 1471-2105-10-152-S7.pdf]
